# Vitamin G: effects of green space on health, well-being, and social safety

**DOI:** 10.1186/1471-2458-6-149

**Published:** 2006-06-07

**Authors:** Peter P Groenewegen, Agnes E van den Berg, Sjerp de Vries, Robert A Verheij

**Affiliations:** 1 NIVEL – Netherlands Institute for Health Services Research, POBox 1568 NL-3500 BN Utrecht, The Netherlands; 2 Utrecht University, Department of Human Geography, Department of Sociology, POBox 80115 NL-3508 TC Utrecht, The Netherlands; 3 Alterra, Green World Research, POBox 47 NL-6700 AA Wageningen, The Netherlands; 4 Wageningen University, Department of Socio-spatial Analysis, POBox 9101 NL-6700 HB Wageningen, The Netherlands

## Abstract

**Background:**

Looking out on and being in the green elements of the landscape around us seem to affect health, well-being and feelings of social safety. This article discusses the design of a research program on the effects of green space in the living environment on health, well-being and social safety.

**Methods/design:**

The program consists of three projects at three different scales: at a macro scale using data on the Netherlands as a whole, at an intermediate scale looking into the specific effect of green space in the urban environment, and at micro scale investigating the effects of allotment gardens. The projects are observational studies, combining existing data on land use and health interview survey data, and collecting new data through questionnaires and interviews. Multilevel analysis and GIS techniques will be used to analyze the data.

**Discussion:**

Previous (experimental) research in environmental psychology has shown that a natural environment has a positive effect on well-being through restoration of stress and attentional fatigue. Descriptive epidemiological research has shown a positive relationship between the amount of green space in the living environment and physical and mental health and longevity.

The program has three aims. First, to document the relationship between the amount and type of green space in people's living environment and their health, well-being, and feelings of safety. Second, to investigate the mechanisms behind this relationship. Mechanisms relate to exposure (leading to stress reduction and attention restoration), healthy behavior and social integration, and selection. Third, to translate the results into policy on the crossroads of spatial planning, public health, and safety. Strong points of our program are: we study several interrelated dependent variables, in different ordinary settings (as opposed to experimental or extreme settings), focusing on different target groups, using appropriate multilevel methods.

## Background

The briefest summary of our program is in its title: Vitamin G, where G stands for the green space around us. Notions of beneficial effects of nearby green space have persisted throughout history [1]. Research on this topic, mainly experimental research, has focused on demonstrating the relationship between exposure to green environments and well-being [2,3]. Most of the evidence on health benefits comes from laboratory experiments that exposed participants to photographic simulations of various types of natural environments [4], or controlled field studies that compared residents with a view of urban greenery to residents without such view [5]. This research has demonstrated that mere exposure to views of nature can improve people's health and well-being by providing restoration from stress and mental fatigue. Moreover, this research has shown that views of nature can improve feelings of neighborhood safety and even lead to decreases in aggression and crime rates [6,7]. To maximize effects, scientists have selected extreme settings, such as urban areas with nearly no green space at all, and concentrated on stress reduction and attention restoration as the most noticeable outcomes. Theoretical developments have followed this focus, and the dominant theories in the field [8,2] consider stress reduction and restoration as a central causal mechanism. Although this focus on extreme settings and restorative effects has highlighted the importance of green space to well-being, it potentially obscures the scope and underlying mechanisms of these effects. Very little is known about the positive effects of green space on well-being through mechanisms of increased and prolonged physical activity, and improved social cohesion [9,10]. Not only causal mechanisms might explain the effects of green space; research in naturalistic settings also has to take into account the possibility of direct or indirect selection [11,12].

Vitamin G aims to establish the relationship between the amount and type of green space in people's living environment and their health, well-being, and feelings of safety, to study the mechanisms behind this relationship, and to specify the implications for policy making. Our program differs from previous studies in two respects.

First of all, field studies will be conducted instead of experimental studies; this is especially important for applied purposes, because it provides a better indication of the relative size of the effects in real-life settings. Field studies also have higher ecological validity, a more direct social relevance, and a focus on long-term rather than on short-term effects (almost inevitable in the case of laboratory studies). Secondly, the focus is on ordinary settings instead of 'extreme' settings in which people are especially stressed or frustrated (hospitals, prisons), or live in extremely poor or barren circumstances (as in some of the previous studies: [13-15]). This increases the generalizability of the effects and the relevance to the European and especially the Dutch situation.

In this article we describe the research questions and background of the program as a whole and the design and methodology of the three separate projects that form the program. Vitamin G started on 1 January 2005 and will run for four years.

### The program vitamin G

The general problem formulation of our research program is: what is the direction and strength of the relationship between the amount of green space in people's living environment and their health, well-being and perceived safety, how can this relationship be explained and how can the results be made useful for policy intervention? This general question will be answered in three projects at three different scales: at a macro scale using data on the Netherlands as a whole, at an intermediate scale looking into the specific effect of green space in the urban environment, and at micro scale investigating the effects of allotment gardens. The specific problem formulations for each of these three projects are:

1. How strong is the relationship between the amount of green space in people's living environment and their perceived health and well-being, and feelings of safety and is this relationship stronger for specific population segments and/or types of green space? How can this relationship be explained?

2. Do urban neighborhoods that differ in the amount and type of green space in the vicinity, also differ in the health, well-being and perceived safety of the people living in these neighborhoods? Have urban neighborhoods that went through a large change in the amount of green space, changed in these respects? If so, which aspects of the green space seem to be the most influential ones?

3. Is having an allotment garden related to health, well-being and perceived safety in urban dwellers and how can this relationship be explained?

**Figure 1:**
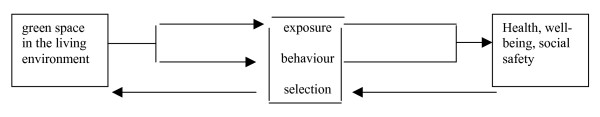
General mechanisms to explain relationships between green space and health, well-being and social safety.

Our approach to answer these questions is based on analyzing the multilevel relationships between environment and people [16,17]. People live in a shared environment that influences their well-being in a general sense, even partly by virtue of the fact that it is a shared environment (as in the case of social integration). These relationships have been schematized in figure 1.

*Exposure *to green space consists of direct physical exposure and the psychological processes through which exposure influences health and well-being. These psychological processes will be further developed, using theories about stress and restoration [4]. Restorative effects can be achieved by merely looking at nature or natural elements, indicating that the aesthetic experience of nature may play a role in this mechanism. Besides providing relief from stress, an aesthetically attractive living environment may also improve well-being by enhancing satisfaction, attachment, and a sense of responsibility. Related to stress reduction, (American) evidence suggests that exposure to natural environments may reduce feelings of anger, frustration and aggression (e.g., [6]). In turn, this may enhance feelings of social safety, and even reduce actual rates of aggressive behavior and criminal activity [7]. Physical exposure to cleaner air may play a role also. Traffic density seems to be the most important source of polluted air in the direct vicinity, while the overall level of air pollution is rather constant in The Netherlands [18].

The *behavioral mechanism *will be developed, using sociological theories about life style, combining structural aspects (socio economic status) and opportunities (availability, social integration) and choices people make (behavior) [19,20]. Natural environments are perceived as more attractive than built environments. Because of this, green areas may stimulate residents to undertake healthy physical activities such as walking or cycling or to choose these activities as a mode of transport, and to spend more time in them [21]. Attractive green areas in the neighborhood may serve as a focal point of tacit coordination for positive informal social interaction, strengthening social ties and thereby social cohesion [15]. Social cohesion by itself is thought to have a positive effect on well-being and feelings of safety. It is important to distinguish the effect of green space from that of population density or urbanicity, which has an established relationship with (mental) health [22].

Apart from these causal mechanisms, part of the effect may be the result of *selection*. Selective migration to or retention in particular living environments might explain part of the relationship between green space in people's living environment and their health. Direct selection occurs, when people's well-being influences their chances of living in a favorable environment; indirect selection, when people with certain characteristics, such as a high income, that are related to well-being can afford to live in a favorable environment [23]. Migration flows in general are related to such socio-demographic characteristics as age, income and education [24]. Consequently, indirect selection might play a role in explaining relationships between the amount of green space in people's living environment and health and well-being. It is therefore important to take into account and control for the possibility of selection.

The three questions formulated above, will be answered in three projects.

The first question will be addressed in a macroscopic project, establishing the strength of the relationships and testing hypotheses about the mechanisms that explain the association between green space and health, well-being, and feelings of safety. Recent epidemiological research by our team has shown a relationship between a green living environment and perceived health indicators in a large population sample [25]. This was the first study in the general population, showing that this relationship was not exclusive to extreme and controlled settings. People living in greener areas tend to perceive their physical and mental health status as better than their counterparts living in less green areas (controlling for socio-economic and demographic spatial clustering). Whether such a positive relationship will also be found when looking at other health indicators is not known. The same applies to people's feelings of safety, which could even be negatively influenced by the presence of green space in one's living environment (because the lack of social control may turn urban green spaces into 'hot spot' for criminal activities).

The second project, addressing the second question, zooms in on the urban environment. Green space is scarcer in urban areas and access to it might be more skewed. Several studies have demonstrated positive relationships between the presence of greenery in urban neighborhoods and residents' health, well-being, and social safety [25,5-7,26]. These relationships have been explained mostly through the mechanism of stress reduction. Indeed, there is some evidence that exposure to local greenery in an urban context may reduce stress and mental fatigue. For example, Honeyman [27] found that stressed participants who viewed images of vegetated urban scenes showed the highest levels of stress reduction, even higher than those viewing countryside, while those viewing barren urban scenes exhibited an increase in stress levels. However, besides stress reduction, there may be other mechanisms underlying beneficial effects of local greenery, in particular increased physical activity and improved social cohesion.

The third project, addressing the third question, focuses on the micro geographic scale. Being also located in an urban environment, it studies people with and without an allotment garden. There is a long history of the use of gardens to improve psychological well-being and physical health [28]. However, few studies have systematically investigated the health benefits of gardens in general, and allotment gardens in particular. Allotment gardens originated at the turn of the 20^th ^century and have known revivals during and after the two world wars to increase supplies of fresh foods [29]. Today, food production is only one of the many functions of allotment gardens. These gardens are now generally assumed to contribute to a wide array of public health and livability issues [30]. Beneficial effects of allotment gardens have been attributed to various factors, including enhanced physical activities, reduced levels of stress and mental fatigue, and a better social and cultural integration [31,32]. Several studies have investigated physical activities associated with gardening [33,34]. In one study among elderly men in The Netherlands, participants spent a greater amount of time per week doing gardening than doing other activities such as walking or cycling [35]. Gardening activities have typically been related to specific health benefits such as reduced cholesterol levels [36]. But there is some evidence that activities on allotment gardens may also contribute to health and well-being in a more general way [37]. When they are asked to describe their reasons for participating in an allotment garden, people often refer to the stress reducing effects of gardening [37]. It has been suggested that in addition to promoting physical activity and reducing stress, allotment gardens may also help to establish a sense of social and cultural integration among gardeners [38]. Especially for older people, allotment gardens may provide a supportive environment that combats social isolation and contributes to the development of their social networks [37].

## Methods/Design

Methods and design will be discussed for each of the three projects that comprise the program separately.

### Vitamin G1: natural environments – healthy environments. exploring the mechanisms

Starting point for the analyses is the positive relationship between green space and people's health that was found by De Vries et al. [25]. In the first step of our analyses we will attempt to replicate the analyses of De Vries et al. using larger, more recent, and more comprehensive datasets that are better tuned to each other. The second step entails the theoretical analysis of the mechanisms responsible for the relationship between people's living environment and their health and well-being. This will result in a number of hypotheses, relating to specific segments of the population, specific types of green space and specific health outcomes, which will subsequently be tested empirically, using (and combining) existing data sets.

Table 1 lists the datasets involved in the analyses. The analyses on health and well-being will be conducted on two datasets that were collected in 2001. The first dataset contains information on perceived general health of about 300.000 people (single question indicator [39]) and can be linked to diagnose-coded contacts with general practice during one year. This large number of subjects guarantees a sufficient power to differentiate between relatively small subgroups in the population. The second dataset contains a much larger set of indicators of health and well-being (acute complaints, chronic illness, mental health, disabilities), health behavior, and socio-economic and demographic variables but on a smaller number of people (N = 13.000). These two datasets do not contain information on feelings of safety. For this particular part of the project we will use data from a population survey on safety and crime (the so-called Politiemonitor [40], N = 90.000). The three individual level datasets can be geographically linked to the fourth dataset, containing information on land use in each 25 by 25 meter gridcell in the Netherlands.

GIS techniques will be used to link the individual level data to the land use data and construct the core independent variables (for example the total amount of green space in a 1 and 3 km radius around one's home). Because spatially clustered data (individuals in their environments) are involved, multilevel research techniques will be used to take into account the hierarchical structure of the data when estimating parameters [41-43].

### Vitamin G2: effects of greenery in urban neighborhoods on health, well-being and social safety

We will apply a twofold methodology: a longitudinal study based on existing neighborhood data and a cross-sectional study based on primary data collection. For the longitudinal study, we will compare data of municipal health services (GGD's) collected prior to and after a considerable change in the local green structure of residential areas. The selection of neighborhoods for the longitudinal study will strongly depend upon the availability of GGD-data on health for neighborhoods that experienced a substantial change in their local greenery situation, prior and after this change. The longitudinal analysis will be rather coarse, since only figures at the level of a residential area as a whole will be available. Furthermore, besides the local greenery situation other characteristics may have changed over time.

The cross-sectional study is more detailed in nature. About eight large Dutch cities will be selected, having a comparable level of urbanity. Within each of these cities about ten neighborhoods will be selected with (as much as possible) a homogeneous and similar population, to diminish the possibility of strong selection effects and to enable comparison of people with similar social-economic characteristics across local greenery situations. The neighborhoods will have to differ on the set of local greenery characteristics that are considered relevant. Based on a review of the literature the most important aspects of the local greenery for each of the proposed mechanisms will be identified (e.g. amount, structure, type, design, maintenance). The inventory of local urban greenery will include site visits and contacts with municipalities of the cities at hand (green department, health department, recreation department, police). GIS-analysis will be used to quantify local greenery characteristics.

Data will be collected by means of a postal self-administered questionnaire (see table 2).

The way questions are posed will be coordinated with those in the other two projects (same phrasing etc.). The same validated measurement scales will be used regarding health as in Vitamin G1. To aid in the identification of green areas, a detailed map will be included in the questionnaire.

Given that the data concern observational units at different levels (individual, neighborhood), the data will be analyzed using multilevel techniques [41-43]. About 80 observational units at the second level (that of the neighborhood) are required to estimate effects of neighborhood characteristics and cross-level interactions [44]. Within each neighborhood about 100 addresses will be randomly selected to participate. Given an expected response rate of 30%, this should result in 30 filled-in questionnaires per neighborhood.

### Vitamin G3: health benefits of allotment gardens

This project focuses on individual residents of deprived urban neighborhoods who spend considerable amounts of time in allotment gardens. The allotment gardeners will be questioned with respect to the same health-related perceptions and behaviors that will be studied in the other two projects. In addition, data will be collected on relevant background variables, such as housing condition and leisure activities. Data will be collected using a mixed methodology of semi-structured face-to-face interviews and the completion of standard weekly diaries over a prolonged period of time (see table 3). Diaries have the advantage of being able to measure exposure time, activities, and mental states in more detail than questionnaires at one point in time. Compared to the other two projects, these data collection methods offer the advantage of gaining detailed insight into the emotional, physical, and spiritual experiences of the gardeners and the factors influencing these experiences.

The selection of appropriate control groups is of critical importance in this project. Ideally, the control group should be comparable to the allotment gardeners in every respect, except for the time spent in an allotment garden. Because this is difficult to realize in practice, we will use two different control groups, each with its own advantages and disadvantages. The first control group consists of close neighbors of the allotment gardeners. Because most deprived neighborhoods in The Netherlands consist of similar row houses or apartments, the members of this control group are likely to be similar to the allotment gardeners with respect to their housing circumstances and other variables. However, self-selection may constitute a problem with this control group, since individuals are more likely to rent allotments when they are in good physical condition. To control for self-selection, we will also compare the allotment gardeners with future allotment gardeners who are on a waiting list for the same gardening complex.

Because of the time-consuming nature of the data collection, this project will include fewer respondents than the other two projects. A feasible design would include 80 gardeners from four different complexes, systematically varying in size (small vs. large) and gardening philosophy (productive vs. recreational). Each gardener will be matched on age, gender, ethnic background and major health risks (e.g., smoking, drinking) to either a neighbor, or a person from the waiting list. This design results in a total sample of 160 respondents, divided in three groups (80 allotment gardeners, 40 neighbors, and 40 future gardeners on a waiting list). Assuming that effect sizes will be medium (0,5), this design provides a power of over 90% at an alpha of 0.05 for detecting differences between the gardeners and the control groups if the two control groups can be combined, and a power of 72% at an alpha of 0.05 for detecting differences between the gardeners and each of the two control groups separately.

An interviewer at home will interview each respondent personally, using a combination of closed and open-ended questions. The formulation of questions will be coordinated with those in the other two projects as much as possible. In as far as possible, validated measurement scales will be used. In addition, gardeners will also be asked to keep a weekly diary for a month. The diary will ask structured questions concerning the gardeners' health and well-being, time spent and activities at the allotment over the previous week, with additional unstructured space in which the respondents are encouraged to discuss events over the week that may have influenced their health, and their thoughts and feelings concerning these events.

## Discussion

Urban green space is under strong pressure [25]. Due to increasing urbanization, combined with a spatial planning policy of densification, more people face the prospect of living in less green residential environments. Especially people from low economic strata, without resources to move to greener areas outside the cities, will be affected. This may lead to environmental injustice with regard to the distribution of (access) to public green space. Until now, the possible effects of these developments on public health and well-being have not been explicitly incorporated in Dutch policy making (see also the advice of the Council for Rural Areas [46]. Policy makers tend to view green space more as a luxury good than as a basic necessity, and appear to overlook the potentially important effects of green space on health, well-being, and safety. It is vital that these findings become implemented in urban planning and design. At present, however, there is not sufficient knowledge to translate findings into guidelines for urban planning and design. In particular, little is known about the strength of the relationships, possible social differences, and the spatial conditions that promote beneficial effects of nearby nature.

The research program Vitamin G aims to fill up these knowledge gaps. Compared to existing studies strong points are:

- We study several interrelated aspects of human well-being that until now have been studied separately: self perceived health, physical complaints, mental health, and perceived safety.

- We include a large number of different settings so that aspects of well-being can be linked to physical characteristics (amount and type of green space needed, visual quality, lay-out, management).

- Field studies will be conducted instead of experimental studies; this is especially important for applied purposes, because it provides a better indication of the relative size of the effects in real-life settings.

- The focus is on ordinary settings instead of 'extreme' settings in which people are especially stressed or frustrated (hospitals, prisons), or live in extremely poor or barren circumstances.

- A focus on different target groups within society increases the policy relevance.

- Distinguishing between individuals and environments makes it possible to analyze these two levels with appropriate statistical models (multilevel analysis).

- The use of similar dependent measures in the three projects and the macroscopic to microscopic approach enables the comparison and integration of the outcomes of the different projects.

The program also has its weak points.

The studies within the program are mainly cross-sectional. As a consequence, selection effects cannot be excluded, although they can be made less probable by appropriate statistical controls. However, longitudinal studies take much more time and are much more expensive. Especially in Vitamin G1 we use datasets that have been collected for other purposes. This means that some variables have not been measured or have been measured in a less appropriate way. An example of a variable that has not been measured in the existing datasets is differential exposure to greenery. However, in the other two projects where primary data will be collected, this will be measured. Especially Vitamin G2 will provide information on the effects of differential exposure.

The program focuses on the beneficial effects of being in a green environment. However, undeniably there are also potential negative effects. Experimental research has shown that nature, in particular wild and uncontrolled nature, is a powerful and probably genetically determined source of fear and anxiety [47,48]. Moreover, urban green space may provoke (feelings of) social unsafety, and tick bites may make people ill. Partly we will take these issues into account by asking respondents what is (un)attractive in green space and how they feel about urban green space. However, because negative health impacts of nature have received a relatively large amount of attention as compared to beneficial effects, we deem it more important to focus on the latter effects.

The program aims to contribute to spatial and health policy-making. Urban green space is under strong pressure [45]. Due to increasing urbanization, combined with a spatial planning policy of densification, more people face the prospect of living in less green residential environments. Especially groups with a low economic status, who do not have the resources to move to greener areas outside the cities, will be affected by these developments. This may lead to environmental injustice with regard to the distribution of (access) to public green space. Until now, the possible effects of the increasing urbanization and environmental injustice on public health and well-being have not been explicitly incorporated in Dutch policy making. Dutch policy makers tend to view green space more as a luxury good than as a basic necessity, and appear to overlook the potentially important effects of green space on health, well-being, and safety. It seems vital that these findings become implemented in urban planning and design. At present, however, there is not sufficiently known about these effects to translate these findings into guidelines for urban planning and design. In particular, little is known about the strength of the relationships, possible group differences, and the spatial conditions that promote beneficial effects of nearby nature.

## Competing interests

The author(s) declare that they have no competing interests.

## Authors' contributions

All authors participated in the design of the program. PG was the principal applicant of the program as whole and drafted the manuscript. RV is responsible for Vitamin G1; SdV for Vitamin G2 and AvdB for Vitamin G3. All authors read the drafts of the manuscript and approved the final version.

## Pre-publication history

The pre-publication history for this paper can be accessed here:



## Figures and Tables

**Table 1:** Datasets to be linked and used in project Vitamin G1

	Subjects	Variables included	Geo coding	Source
*Dataset 1*. Health and well-being 1	All people listed in 104 GP practices. N = 300.000. Representative for Dutch population.	• perceived general health •basic socio-economic and demographic variables, including ethnic background •all diagnose-coded contacts and interventions with general practice during 12 months in 2000/1.	6-digit postcode	Second National Survey of Morbidity and Interventions in General Practice, 2001 [49]
*Dataset 2*. Health and well-being 2	Random sample of patients listed in 104 GP practices. N = 13.000. Representative for Dutch population. This is a subset of the subjects the first dataset.	as dataset 1, *plus: *• extensive health information •health behavior (e.g. smoking, nutrition, physical activity) •extensive socio-economic and demographic characteristics •other relevant control variables (e.g. having a private garden, housing conditions)	6-digit postcode	Second National Survey of Morbidity and Interventions in General Practice, 2001 [49].
*Dataset 3*. Feelings of safety	Random population sample. N = 90.000	•feelings of safety • perceived neighborhood problems •having been victim of crime •socio-economic and demographic characteristics, incl. ethnicity	4-digit postcode	Politiemonitor Bevolking 2003 [40]
*Dataset 4*. Green space	The Netherlands, 25*25 meter grid cells	land use data (type of green, water surface, built-up area).	x- and y- coordinates grid cells	LGN4 data

**Table 2:** Primary data to be collected in project Vitamin G2 in a 100 person sample in each of 80 urban neighborhoods

1) Primary dependent variables	perceived general health, acute complaints, chronic illness, mental health, disabilities, well-being, feelings of safety
2) Variables related to underlying mechanisms	levels of stress, mental fatigue
	use of local green areas: distance from home, frequency of visitation, duration of visits, activities performed during visits, accompanying persons, interactions with other people during visits
	perceived social cohesion, participation in neighborhood social activities, social contacts
3) Variables related to the supply of urban greenery	detailed land use data, observed greenery situation from site visits
4) Other relevant variables (including possible confounders), such as:	risky (such as smoking, drinking) and positive health behavior (e.g. total amount of physical activity)
	knowledge, perception, and evaluation of green elements within the local environment
	possession of durable consumer goods: allotment garden, caravan, sports equipment and their use
	participation in alternative leisure activities: non-green activities and visits to green areas outside the living environment (distance, frequency, duration, activities), holidays and short breaks
	socio-economic background characteristics of the respondent, including housing conditions

**Table 3:** Design and data collection of project Vitamin G3

Focal group: allotment gardeners	80	quantitative: questionnaire qualitative: open-ended interview, time diary
Control group 1: next door neighbors	40	idem
Control group 2: waiting list for allotment garden	40	idem
